# Nonfunctionalized Cation of an Ionic Liquid as a Ligand in the Synthesis of a New Coordination Compound and Assessment of Its Biological Activity

**DOI:** 10.1155/2019/9257679

**Published:** 2019-06-24

**Authors:** Getinet Tamiru Tigineh, Atakilt Abebe

**Affiliations:** Bahir Dar University, Science College, Chemistry Department, Bahir Dar, Ethiopia

## Abstract

Literature evidences reveal the affinity of ionic liquids for biomembranes that they are readily absorbed into the cell, resulting in a variety of biological effects, including broad antibacterial potential and anticancer activity. Recent research directions considered the ions of this class of compounds as a new choice of ligands in the synthesis of transition metal complexes for various applications. Based on this, the present work reports the synthesis, structural characterization, and *in vitro* antibacterial activities of a tetrahedral hexacationic Co(II) complex formed by coordinating with the cation of an ionic liquid, *N*-butyl-4,4-bipyridinium bis(trifluoromethylsulfonyl)amide ([C_4_Bip][Tf_2_N]). It has been demonstrated by the isolation and characterization of tetrakis-(*N*-butyl-4,4′-bipyridinium)cobalt(II)dichloride-tetrakis-(bis(trifluoromethylsulfonyl)amide, ([(C_4_Bip)_4_Co]Cl_2_(Tf_2_N)_4_). The ligand and complex are characterized spectroscopically (^1^H, ^13^C, and ^19^F NMR, ESI MS, ICP OES), and by CHNS elemental analysis, halide estimation, and conductivity studies. The antibacterial activities of the compounds against two bacteria, *Klebsiella pneumoniae* (*K*. *pneumoniae*) and *Staphylococcus aureus* (*S. aureus*), are screened using the agar well-diffusion method and were compared with a reference (gentamicin). The metal complex demonstrated better inhibition than the ionic liquid and the reference.

## 1. Introduction

Ionic liquids (ILs) are salts with a melting point of less than 100°C. Many of them are liquids at and/or below room temperature and are thus called room temperature ionic liquids (RTILs) [1]. The choice of appropriate cation-anion combinations enables to design an IL with a set of desired properties for a specific application [2]. This makes them a class of materials for a wide spectrum of applications that academia and industries are attracted to [3–5]. Recently, these liquids are attracting increasingly the attention of inorganic and material chemists for the synthesis of zeolites and nanoparticles [6–12], in electrochemical applications such as electrolytes in batteries and in photovoltaic devices, a medium for electrodeposition or electropolishing of metals [13–17], in pharmaceuticals synthesis, and in drug delivery since the ions from this family could represent a new choice of versatile ligands, able to interact with lipids and proteins, affecting the structure, dynamics, and activity of biomembranes [18–20]. The permutation between the large number of cations and anions allows a large number of ionic liquids with a corresponding amount of properties which can offer unique possibilities to the inorganic chemist. For instance, their application in coordination chemistry is important that their coordination tendency to metal ions and the property of the combination need investigation. In this regard, a number of reports disclosed that typical ionic liquids do not extract metal cations because of their poor coordination. Even if they do, the coordination takes place between the metal ion and the poorly coordinating anions of the ionic liquid [21–23]. This required the use of some extractants or functionalized ionic liquids incorporating some functional groups in their cations and/or anions which makes them both solvent and extractant [2, 24]. However, these strategies require further amount of synthetic work and render the final application more laborious, expensive, and less environmental friendly. Thus, the objective of this work was to investigate the coordination ability of a nonfunctionalized cation of an ionic liquid to a metal ion. The outcome of the investigation was a hexacationic coordination compound. The result introduces a promising new material which contains the combined features of ionic liquids and metal ions such as medicinal, magnetic, spectroscopic, and catalytic properties.

## 2. Materials and Methods

4,4′-Bipyridine and 1,4-dioxane were obtained from Sigma-Aldrich; 1-bromobutane and lithium bis(trifluoromethylsulfonyl)amide were purchased from Alfa Aesar. All solvents were purified employing standard drying agents prior to use.

CHNS elemental analyses were made using a 5E-CHN2200 elemental analyzer taking 20 mg sample. ^1^H and ^13^C NMR, using a Bruker AM-270 (270 MHz) and Bruker 400 MHz spectrometers, and ^19^F NMR using a Bruker AV-400 (376.5 MHz) spectrometer were employed to confirm the structures and check the purity of the synthesized ligand salt and complex. ESI MS was used to determine the molecular ion mass of the ligand and complex using Bruker Micro TOF. Bromide and chloride estimation was conducted taking 30 mg sample and dissolving in 40 mL distilled water. Excess AgNO_3_ solution was added for the formation of AgBr and AgCl precipitate. Then, the cruddy white precipitate formed was filtered and dried in an oven, and the amount of bromide and chloride was calculated from the weight difference. Conductivity of 3 × 10^−4^ M ethanolic solution of the complex was also investigated using a Bante901P portable pH/conductivity/TDS meter at room temperature.

### 2.1. Synthesis

**Scheme 1:**
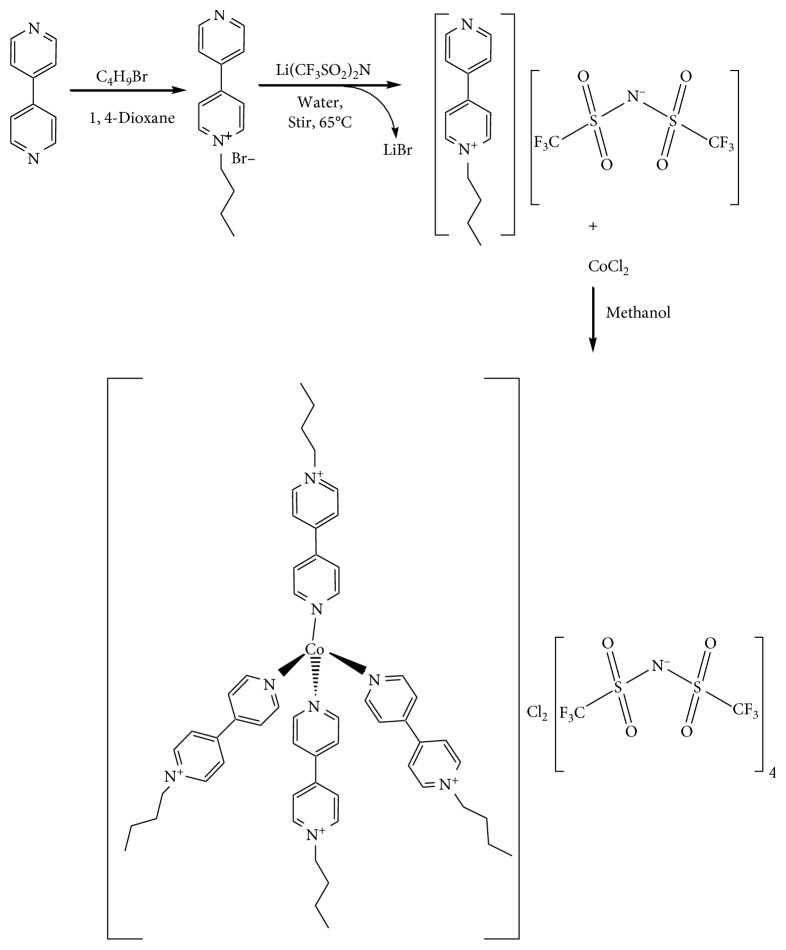
Synthesis strategy of the complex.

*N*-Butyl-4,4′-bipyridinium bromide [C_4_Bip]Br and *N*-butyl-4,4-bipyridinium bis(trifluoromethylsulfonyl)amide [C_4_Bip][Tf_2_N] were prepared following literature report [25] (Scheme 1).

### 2.2. *N*-Butyl-4,4′-bipyridinium Bromide [C_4_Bip]Br

Four grams (0.0256 mol) of 4,4′-bipyridine was dissolved in 30 mL dry 1,4-dioxane in a two-necked 100 mL round-bottomed flask fitted with a condenser. 3.52 g (0.0257 mol, 2.45 mL) of 1-bromobutane dissolved in 10 mL dry 1,4-dioxane was added from a dropping funnel, and the mixture was allowed to stir at 65°C for 24 h and a white precipitate was filtered (yield: 5.48 g, 73%).

### 2.3. *N*-Butyl-4,4′-bipyridinium Bis(trifluoromethylsulfonyl) Amide [C_4_Bip][Tf_2_N]

To a 80 mL aqueous solution of [C_4_Bip]Br (2.5 g, 8.5 mmol) in a double-neck round bottomed flask fitted with a condenser being stirred in an oil bath at 65°C, slightly excess molar equivalent (2.53 g, 8.8 mmol) of Li(Tf_2_N) dissolved in 80 mL of distilled water was added dropwise. Consequently, oily droplets formation was started and the stirring continued for an hour. The mixture was left to stand overnight, and a yellowish separate, denser phase was obtained. The oily phase was decanted and dissolved in methanol and stirred with activated charcoal for 30 min and filtered. A colorless solution was obtained. The methanol was removed under vacuum, and a colorless viscous mass was collected (yield: 3.56 g, 84.73%).

### 2.4. Tetrakis-(*N*-butyl-4,4′-bipyridinium)cobalt(II) Dichloride Tetrakis-(bis(trifluoromethylsulfonyl) Amide [Co(C_4_Bip)_4_]Cl_2_(Tf_2_N)_4_

To a methanolic solution of CoCl_2_ (0.025 g, 1.925 mmol) being stirred magnetically in a water bath at room temperature, a methanolic solution of *N*-butyl-4,4′-bipyridiniumbis (trifluoromethylsulfonyl) amide (0.380 g, 7.702 mmol) was added from a dropping funnel and stirred for 90 min until the addition of the reagent was completed. Then, the mixture was further stirred for 1 h at 35°C. A light pink colored homogenous solution was obtained. The methanol was removed in vacuum. Blue powder was collected and washed three times with acetone to remove any excess *N*-butyl-4,4′-bipyridinium (bis(trifluoromethylsulfonyl) amide. It was recrystallized from methanol to remove any unreacted CoCl_2_ (yield: 0.374 g, 92.3%). The synthesis path is indicated in Scheme 1.

#### 2.4.1. Antibacterial Activity Testing

The ionic liquid (ligand) and its Co(II) complex were evaluated for *in vitro* antibacterial activities against strains of *S. aureus* and *K*. *pneumoniae*. The strains were maintained in the appropriate blood agar base at 4°C. Gentamicin was used as reference. The experiments were repeated three times to obtain consistent results. The antibacterial tests were carried out at Bahir Dar University, Department of Biology, Microbiology Laboratory, Bahir Dar, Ethiopia.

#### 2.4.2. ^1^H, ^13^C, and ^19^F NMR

**Figure 1:**
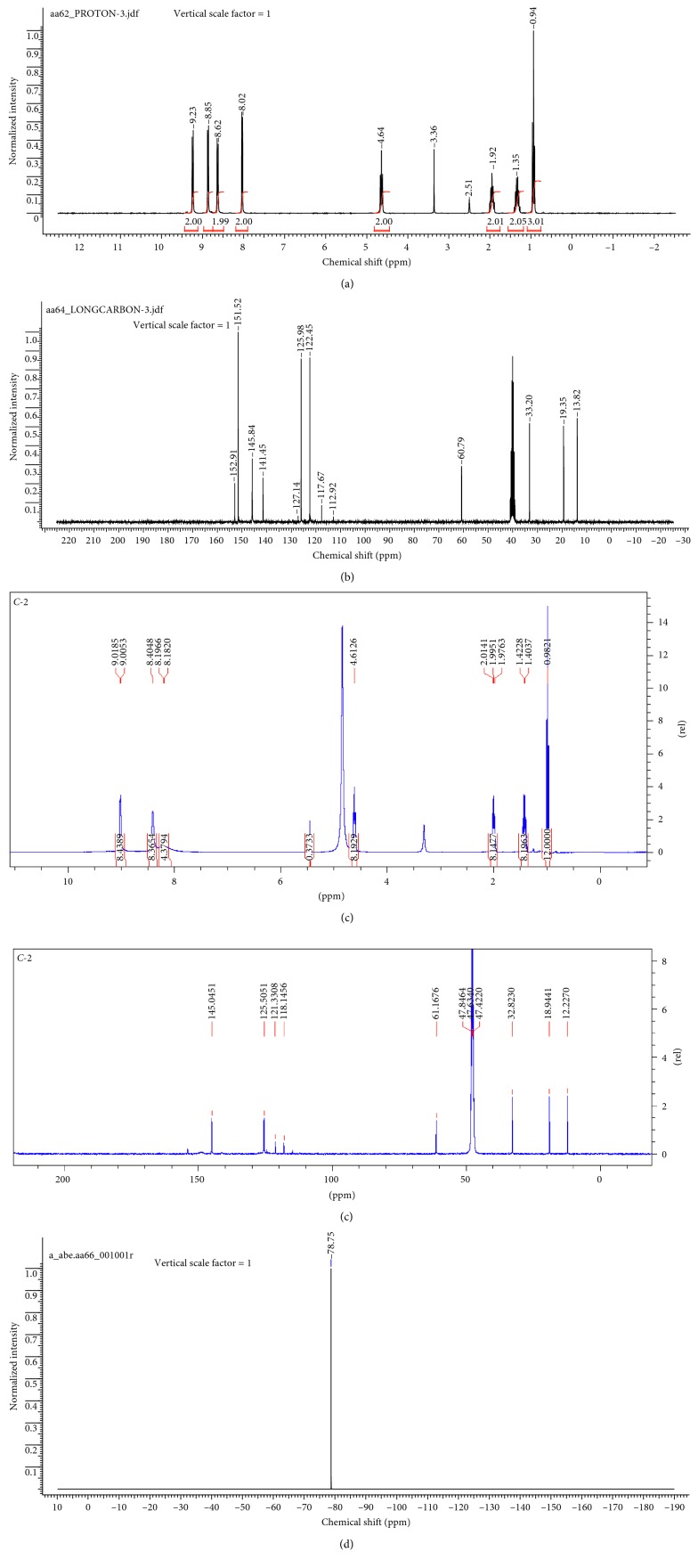
(a) ^1^H NMR of the ionic liquid; (b) ^13^C NMR of the ionic liquid; (c) ^1^H NMR of the complex; (d) ^13^C NMR of the complex; (e) ^19^F NMR of the ligand.

The ^1^H, ^13^C, and^19^F NMR of the ligand and complex are indicated in Figure 1.

## 3. Results and Discussion

**Figure 2:**
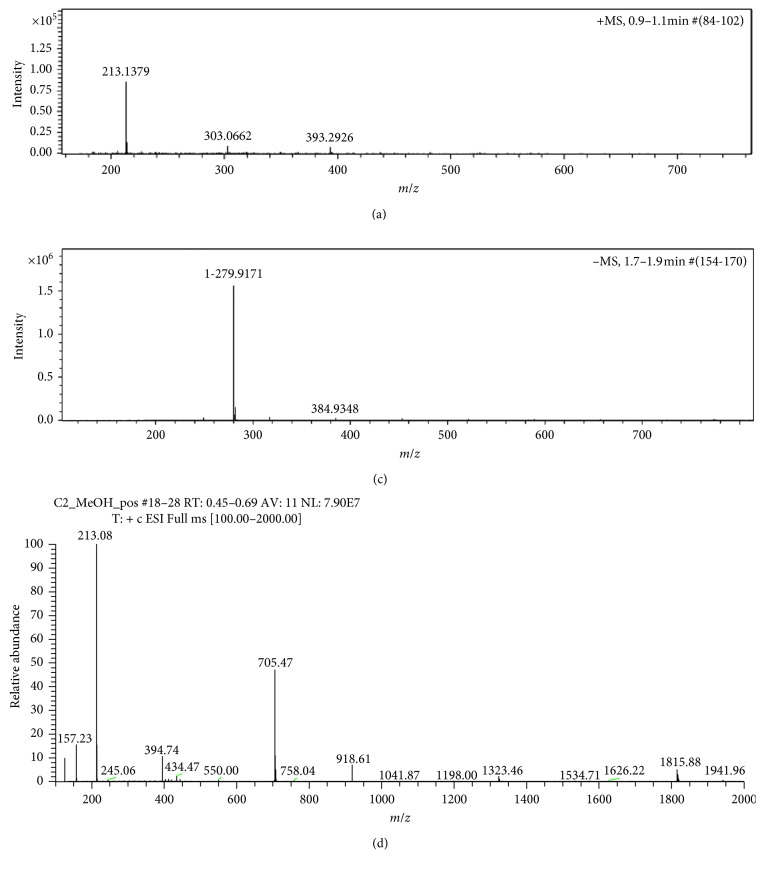
ESI MS of (a) the cation of the ionic liquid, (b) anion of the ionic liquid, and (c) cation of the complex.

The synthesis of the ligand is evident from NMR and ESI MS data (Figures 1(a)–1(e) and 2(a) and 2(b). The appearance of four and eight peaks in ^1^H NMR and ^13^C NMR in the aromatic region, respectively, and the up-field appearance of the appropriate number of peaks representing alkyl protons and carbon are strong confirmations for the occurrence of monoquaternization. Moreover, the molecular positive ion peak (*m*/*z* = 213.1379) obtained from ESI MS spectra confirmed the synthesis of the intended structure (Figure 2(a)). Furthermore, the complete anion exchange performed to acquire pure ionic liquid is also confirmed from the appearance of only one peak at *δ* = −78.75 ppm in the ^19^F NMR (Figure 1(e)) and molecular negative ion peak (*m*/*z* = 279.9171) in the ESI MS of (CF_3_SO_2_)_2_N^−^ (Figure 2(b)). This evidence was compounded with the appearance of quartet peaks in the range *δ* = 112.92–127.14 ppm of ^13^C NMR (Figure 1(b)). The latter is the characteristic of carbon coupled with three fluorine atoms of the exchanged anion ((CF_3_SO_2_)_2_N^−^).

The coordination of the cation of the ionic liquid to Co(II) resulted a hexacationic but lipophilic salt. The formation of the target compound was confirmed by CHNS elemental analysis and Co(II) and halide estimation experiment results. Element found(calculated): C, 36.33(36.54); H, 2.98(3.24); N, 7.54(7.99), S, 11.88(12.18); Co, 2.68(2.80); Cl, 2.89(3.38).

Furthermore, ^1^H, and ^13^C NMR (Figures 1(c) and 1(d)) confirmed the coordination of the cation of the ligand. In the ^1^H NMR spectrum, the number of signals in the aromatic region is reduced due to the influence of the paramagnetic Co(II) on certain protons in the aromatic portion of the ligand [26]. The ESI MS spectrum recorded dissolved in methanol signaled molecular ion peak for the cation (*m*/*z* = 943.22/6 = 157.23) (Figure 2(c)) which shows the coordination of four *N*-butyl-4,4′-bipyridinium and one methanol molecule to Co(II). This is in a very good agreement with the observation that the complex demonstrated pink and blue colors in solution and dried, respectively.

The complex demonstrated significantly lower molar conductivity value in ethanol (124.33 S·mol^−1^·cm^2^) than expected from 1 : 6 cation to anion ratio. The diminished conductivity is attributed to increase in the drifting (counterdirectional) speed. This is due to the strong solute (complex) and solvent (ethanol) interaction with the solvent cavity surrounding the cation and the anion. This could be anticipated due to the lipophilicity of the alkyl chain in the cation. Furthermore, the large molar masses of the cation and the anion retard speed of their motion. Moreover, the large sizes of the cation and the anion increase the frictional force as they move in opposite directions to their corresponding electrodes [27, 28].

**Figure 3:**
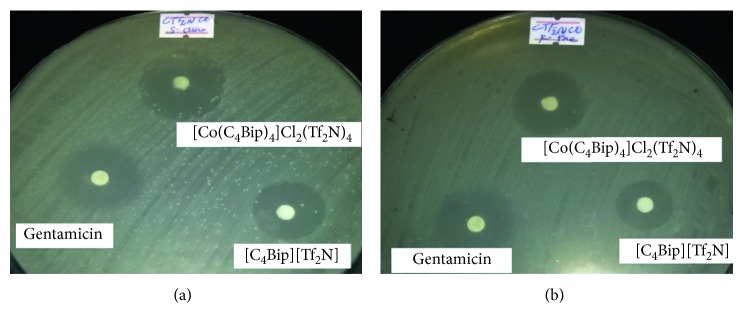
Inhibition observed by the ionic liquid and its complex as well as the reference. (a) Gram-negative bacteria *K*. *pneumoniae*. (b) Gram-positive bacteria *S. aureus*.

The biological activity test shows that the ionic liquid and its Co(II) complex are biologically active against both the tested strains (Figure 3). The coordination of the ionic liquid to the metal enhanced its activity against both strains. This could be anticipated as a result of the formation of rigid configuration of the ligand following the coordination. Compared with the reference compound gentamicin, the present complex is more active by 6.2% and 10% against *K*. *pneumoniae* and *S. aureus*, respectively (Table 1).

## 4. Conclusions

The studies on the elemental analysis and ESI MS among other methods indicate the formation of a hexapositively charged hydrophobic complex by coordination of four cations of the ionic liquid with Co(II). The electrical conductance study in ethanol indicated the low electrolytic nature of the complex regardless of the large number of ions as a consequence of strong solvent-complex interactions, strong cation-anion friction, and significantly large mass of the cation and anion. The investigations on antimicrobial activity indicate the increased activity of the ionic liquid while coordinated to Co(II) as a consequence of improvement of the penetration of the complex into the lipid membrane and interference in the normal activity of the bacteria. The improved activity of the complex against both Gram-negative and Gram-positive bacteria indicates its wide range activity.

## Figures and Tables

**Table 1:** Inhibition zones of the ionic liquid, the complex, and the reference.

	Inhibition zone (mm)	
Compound	Gram-negative bacteria *K*. *pneumoniae*	Gram-positive bacteria *S. aureus*
[C_4_Bip][Tf_2_N]	21.00 ± 0.03	27.00 ± 0.33
[Co(C_4_Bip)_4_]Cl_2_(Tf_2_N)_4_	31.86 ± 0.24	33.00 ± 0.23
Gentamicin	30.00 ± 0.32	30.00 ± 0.34

## Data Availability

The data used to support the findings of this study are included within the article.

## References

[1] Mandai T., Yoshida K., Ueno K., Dokko K., Watanabe M. (2014). Criteria for solvate ionic liquids. *Physical Chemistry Chemical Physics*.

[2] Nockemann P., Pellens M., Van Hecke K. (2010). Cobalt(II) complexes of nitrile-functionalized ionic liquids. *Chemistry—A European Journal*.

[3] Angell C. A., Ansari Y., Zhao Z. (2012). Ionic liquids: past, present and future. *Faraday Discussions*.

[4] Shamshina J. L., Wang H., Zhou X., Gurau G., Rogers R. D., Zhang W., Cue B. W. (2018). Ionic liquids in pharmaceutical industry. *Green Techniques for Organic Synthesis and Medicinal Chemistry*.

[5] Liwarska-Bizukojc E., Maton C., Stevens C. V. (2015). Biodegradation of imidazolium ionic liquids by activated sludge microorganisms. *Biodegradation*.

[6] Smiglak M., Pringle J. M., Lu X. (2014). Ionic liquids for energy, materials, and medicine. *Chemical Communications*.

[7] Yuan D., He D., Xu S. (2015). Imidazolium-based ionic liquids as novel organic SDA to synthesize high-silica Y zeolite. *Microporous and Mesoporous Materials*.

[8] Zhou Y., Antonietti M. (2003). Preparation of highly ordered monolithic super-microporous lamellar silica with a room-temperature ionic liquid as template via the nanocasting technique. *Advanced Materials*.

[9] Nakashima T., Kimizuka N. (2003). Interfacial synthesis of hollow TiO_2_ microspheres in ionic liquids. *Journal of the American Chemical Society*.

[10] Drylie E. A., Wragg D. S., Parnham E. R. (2007). Ionothermal synthesis of unusual choline-templated cobalt aluminophosphates. *Angewandte Chemie*.

[11] Correa C. M., Faez R., Bizeto M. A., Camilo F. F. (2012). One-pot synthesis of a polyaniline–silver nanocomposite prepared in ionic liquid. *RSC Advances*.

[12] Ahmed E., Breternitz J., Groh M. F., Ruck M. (2012). Ionic liquids as crystallisation media for inorganic materials. *CrystEngComm*.

[13] MacFarlane D. R., Tachikawa N., Forsyth M. (2014). Energy applications of ionic liquids. *Energy & Environmental Science*.

[14] Miran M. S., Kinoshita H., Yasuda T., Susan M. A. B. H., Watanabe M. (2012). Physicochemical properties determined by ΔpKa for protic ionic liquids based on an organic super-strong base with various Brønsted acids. *Physical Chemistry Chemical Physics*.

[15] Srour H., Rouault H., Santini C. (2013). Imidazolium based ionic liquid electrolytes for Li-ion secondary batteries based on graphite and LiFePO_4_. *Journal of the Electrochemical Society*.

[16] Cao Y., Zhang J., Bai Y. (2008). Dye-sensitized solar cells with solvent-free ionic liquid electrolytes. *Journal of Physical Chemistry C*.

[17] Bakkar A., Neubert V. (2015). A new method for practical electrodeposition of aluminium from ionic liquids. *Electrochemistry Communications*.

[18] Ferraz R., Branco L. C., Prudêncio C., Noronha J. P., Petrovski Ž. (2011). Ionic liquids as active pharmaceutical ingredients. *ChemMedChem*.

[19] Marrucho I. M., Branco L. C., Rebelo L. P. N. (2014). Ionic liquids in pharmaceutical applications. *Annual Review of Chemical and Biomolecular Engineering*.

[20] Benedetto A., Ballone P. (2018). Room-temperature ionic liquids and biomembranes: setting the stage for applications in pharmacology, biomedicine, and bionanotechnology. *Langmuir*.

[21] Kuzmina O., Hassan N. H., Patel L. (2017). The impact of ionic liquids on the coordination of anions with solvatochromic copper complexes. *Dalton Transactions*.

[22] Zhou Y., Boudesocque S., Mohamadou A., Dupont L. (2015). Extraction of metal ions with task specific ionic liquids: influence of a coordinating anion. *Separation Science and Technology*.

[23] Williams D. B., Stoll M. E., Scott B. L., Costa D. A., Oldham W. J. (2005). Coordination chemistry of the bis (trifluoromethylsulfonyl) imide anion: molecular interactions in room temperature ionic liquids. *Chemical Communications*.

[24] Olivier J.-H., Camerel F., Selb J., Retailleau P., Ziessel R. (2009). Terpyridine-functionalized imidazolium ionic liquids. *Chemical Communications*.

[25] Abebe A., Admassie S., Villar-Garcia I. J., Chebude Y. (2013). 4,4-Bipyridinium ionic liquids exhibiting excellent solubility for metal salts: potential solvents for electrode position. *Inorganic Chemistry Communications*.

[26] Bertini I., Messori L., Golub G., Cohen H., Meyerstein D. (1995). A 1H NMR study of the complex of cobalt (II) with 2, 5, 8, 11-tetramethyl-2, 5, 8, 11-tetraazadodecane in aerated aqueous solutions. *Inorganica Chimica Acta*.

[27] Bonchio M., Carraro M., Casella G., Causin V., Rastrelli F., Saielli G. (2012). Thermal behaviour and electrochemical properties of bis(trifluoromethanesulfonyl)amide and dodecatungstosilicate viologen dimers. *Physical Chemistry Chemical Physics*.

[28] Atkins P. W. (1994). *Physical Chemistry*.

